# Imperfections Formation in Thin Layers of NiTi Triply Periodic Minimal Surface Lattices Fabricated Using Laser Powder Bed Fusion

**DOI:** 10.3390/ma15227950

**Published:** 2022-11-10

**Authors:** Shahadat Hussain, Ali N. Alagha, Wael Zaki

**Affiliations:** 1 Advanced Digital and Additive Manufacturing Center, Khalifa University of Science and Technology, Abu Dhabi P.O. Box 127788, United Arab Emirates; 2 Department of Mechanical Engineering, Khalifa University of Science and Technology, Abu Dhabi P.O. Box 127788, United Arab Emirates

**Keywords:** shape memory alloys, additive manufacturing, triply periodic minimal surfaces, architected materials, laser powder bed fusion

## Abstract

In this paper, thin layers of NiTi shape memory alloy (SMA) triply periodic minimal surface lattices (TPMS) are fabricated using laser powder bed fusion (LPBF), considering different laser scanning strategies and relative densities. The obtained architected samples are studied using experimental methods to characterize their microstructural features, including the formation of cracks and balling imperfections. It is observed that balling is not only affected by the parameters of the fabrication process but also by structural characteristics, including the effective densities of the fabricated samples. In particular, it is reported here that higher densities of the TPMS geometries considered are generally associated with increased dimensions of balling imperfections. Moreover, scanning strategies at 45° angle with respect to the principal axes of the samples resulted in increased balling.

## 1. Introduction

NiTi shape memory alloys belong to the class of smart materials with a wide range of applications in the field of biomedical [1], aerospace [2], robotics [3], and automotive engineering [4]. Among other SMAs, NiTi remains the most widely used, owing to its high actuation energy density [5], superior functional properties, high ductility, low corrosion rate [6], biocompatibility [7], and high damping ratio [8]. Additive manufacturing (AM) has recently gained popularity in relation to the fabrication of NiTi intricate parts [9,10,11,12] because it naturally overcomes long-standing issues with conventional NiTi manufacturing techniques, including poor weldability [13] and machinability [14]. In comparison with other AM techniques, such as direct energy deposition [15], wire and arc additive manufacturing [16], laser powder bed fusion (LPBF) can provide better dimensional accuracy, generally denser parts, and the ability to fabricate more complex geometries with better surface finish [15]. Consequently, LPBF is one of the most utilized AM techniques for fabricating architected parts made of NiTi [17,18,19] as well as many other materials [20,21,22]. Architected porous NiTi constructs are attractive because they achieve low specific weight [23] combined with high specific stiffness and strength [24,25,26]. Moreover, porous/cellular structures may well be engineered for good fluid permeability [27,28], enhanced heat dissipation [29], and improved electrical properties [30]. They may further be used for artificial tissue engineering [31] and impact energy absorption [32]. Among porous structures, those featuring triply periodic minimal surface (TPMS) architectures have been gaining increased attention in parallel with the democratization of AM solutions [33,34,35]. These periodic three-dimensional architectures feature zero mean curvature and allow the segregation of space into two or more intertwined but non-connecting sub-domains. Moreover, TPMS structures have a large surface-to-volume ratio and are free of self-intersections. 

In the literature, very few references [36,37,38,39] have addressed the fabrication of NiTi TPMS structures. In these papers, the properties of the printed TPMS structures are found to be significantly affected by fabrication process parameters, such as laser power, scan speed, energy density, layer thickness, hatch spacing, and scan strategy. The influence of scan strategy on the outcome of LPBF fabrication has received relatively little attention compared to parameters, such as laser power, scanning speed, hatch spacing, and layer thickness, which influence the rate at which energy is supplied to the powder bed. In this respect, Amirjan et al. [40] reported that rotating the scan vector between successive layers was more advantageous to obtain uniform temperature distribution during AM. Such rotation was found to gradually alter the direction of heat flux in-between layers, resulting in a more uniform temperature distribution. Therefore, the scan strategy was proposed to have the potential for printing isotropic materials with better mechanical properties. In contrast, the island scanning strategy, fractal scanning strategy, and spiral/helix scanning strategy usually involve shorter scan vectors. It was observed [41,42,43] that shorter scan vector lengths may help reduce residual stress and, thereby, improve mechanical properties. Ali et al. [44] highlighted an improvement in superelasticity in tension in SMAs due to alternating x/y axes scanning strategies. It was further concluded that scanning strategies played a significant role in microstructural evolution during solidification and thereby affecting mechanical as well as functional properties in SMAs. Kruth et al. [45] found that the greatest residual stress was developed perpendicular to the scan direction. However, other studies [46,47] contradicted the former finding and observed that the greatest stress was generated parallel to the scan direction. Ramos et al. [48] confirmed the influence of scanning strategies on residual stresses and deformation of the component and observed lower residual stress generation due to shorter scan vector length. It was also observed that sequences of scan vectors proved more effective in reducing the deformation of printed parts. In parallel, a combination of lower hatch spacing, higher power, and scan speed or, alternatively, moderate power and lower scan speed, was found to increase the density of AM fabricated parts. 

In the present study, the influence of relative sample density and laser scanning strategy on the microstructure of NiTi TPMS layers is investigated for the first time. The results help address a gap in current research in relation to the influence of process parameters and structural design features on the behavior of NiTi TPMS lattices.

## 2. Materials and Methods

### 2.1. NiTi Powder

**Figure 1:**
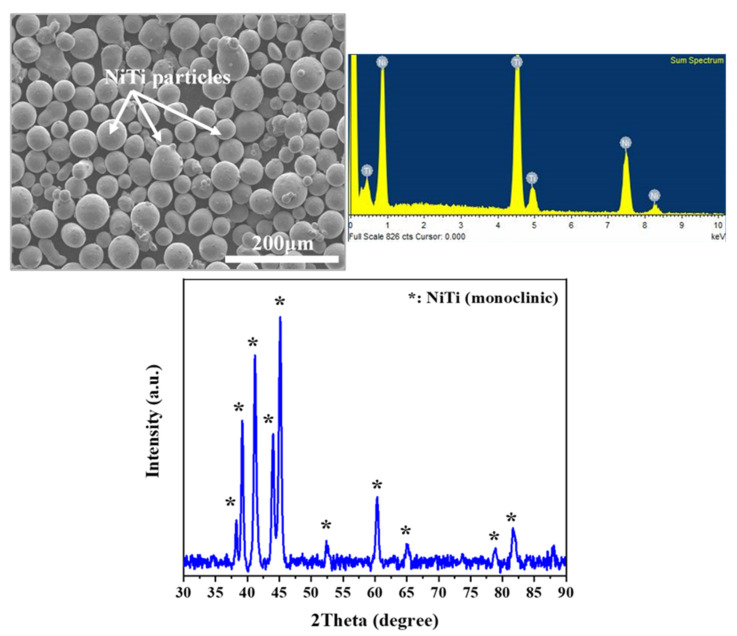
Electron micrograph showing NiTi powder, sum spectrum depicting the presence of nickel and titanium as constituents of the powder, and x-ray diffraction plot of NiTi powder depicting monoclinic intermetallic NiTi phase peaks denoted by *.

Gas-atomized pre-alloyed NiTi powder (Figure 1) was procured from TLS Technik GmbH & Co., Bitterfeld-Wolfen, Germany, with varying average particle sizes between 15 and 46 μm. The spherical particles were argon atomized and were packed in air-tight containers to avoid oxidation. 

### 2.2. Design of TPMS Lattice Structures

In the present study, the Schwartz primitive TPMS topology was selected for the morphological investigation of additively printed structures. The geometries of the samples were generated considering approximate surface equations of the form shown in the Equation (1).

(1) $$\cos\left(x\right)+\cos\left(y\right)+\cos\left(z\right)=c$$

where *x, y, z* are Cartesian coordinates, and *c* is a level-set iso-value constant.

**Figure 2:**
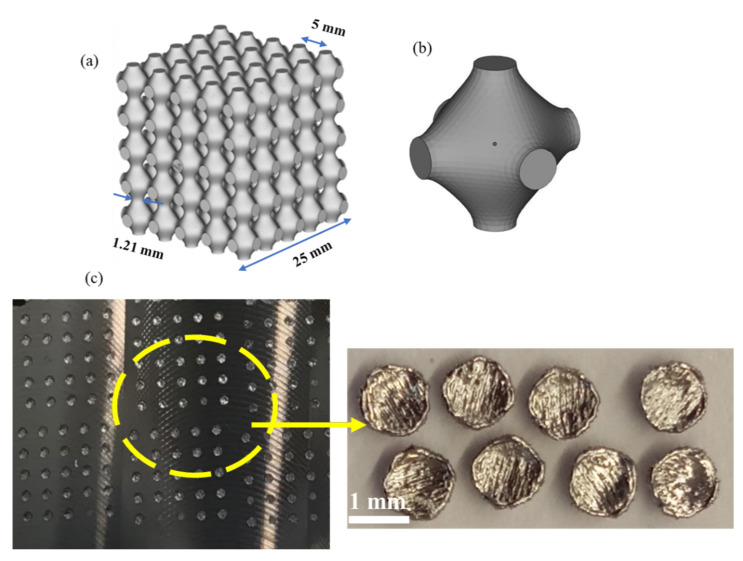
CAD model of the TPMS lattice showing a primitive lattice (**a**) and its corresponding unit cell (**b**). The thin TPMS layer was fabricated on a titanium-alloy base plate (**c**).

MSLattice [49] was employed to plot the TPMS structures, as shown in Figure 2. The samples consisted of cubic Schwartz primitive cells of length 25 mm and relative density in the range of 30% to 60%. The relative density ($\rho_{R}$) is calculated based on the CAD model by dividing the volume filled by the sample by the volume of a cube with similar dimensions (reference cube), as follows (Equation (2)):

(2) $$\rho_{R}=\frac{V_{S}}{V}\;$$

where $V_{S}$ is the volume of the solid part of the cell and V is its total volume. 

The surface area per unit cell of the primitive lattice with 30% relative density was calculated to be 61.1 mm^2^. The surface area plays an important role in relation to the structural heat balance during AM. It affects the melt pool dynamics during solidification, thereby influencing the overall mechanical and functional properties of the fabricated samples. The strut thickness of primitive unit cell was 1.21 mm. The varying strut thickness and its geometry play a significant role during the solidification of the melt.

**Figure 3:**
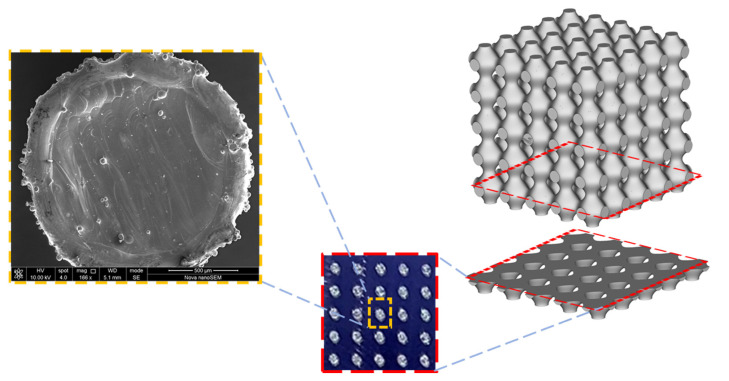
Transverse section of CAD model showing thin layers of printed primitive samples.

Figure 3 further illustrates the sectioning of the CAD model to obtain the studied primitive lattices.

### 2.3. Additive Manufacturing of the NiTi Samples

**Figure 4:**
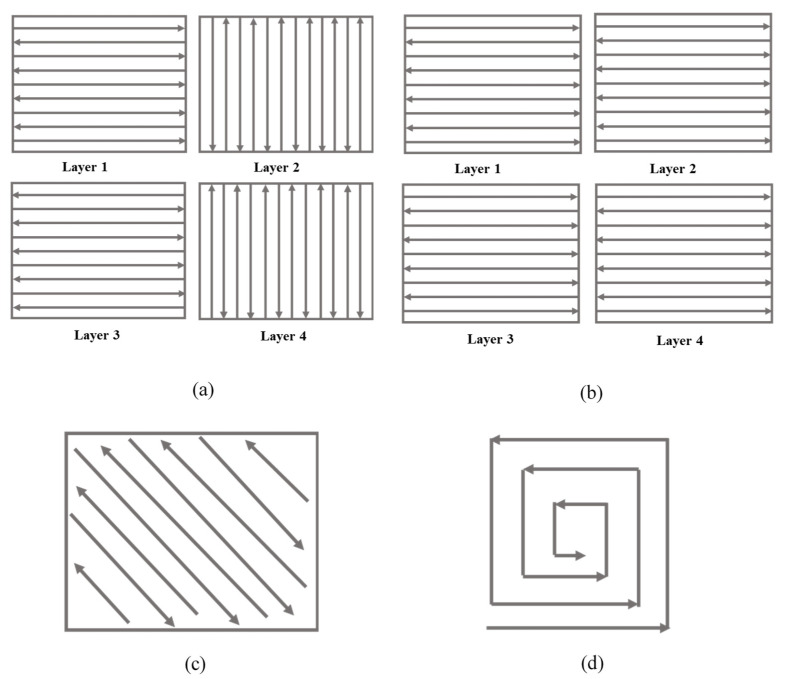
Various scan strategies used for the fabrication of primitive lattice layers: (**a**) Parallel with 90° rotation, (**b**) parallel with 0° rotation, (**c**) inclined at 45°, and (**d**) spiral.

An EOS-M400 3D metal printer was used for additive manufacturing of the NiTi samples. The build chamber was preheated to 85 °C and maintained at the same temperature during fabrication, and a Ti-alloy-based plate was utilized. Preheating to 85 °C was the highest preheating temperature that could be achieved for our experiment with the EOS M400-4. Reaching this temperature took almost 24 h, and it could not be increased further. One issue here is that the printer does not allow for preheating the inert gas flow, which results in convective heat loss in the build chamber. Higher preheating temperatures will likely be beneficial in moderating residual stress fields that may arise during the building process because of severe temperature gradients and metallurgical interactions. The printer has four Ytterbium-fiber lasers of 400 W maximum power each, a scanning speed of up to 7.0 m/s, and a build volume of 400 × 400 × 400 mm^3^. The focus diameter of the laser beam was around 90 μm with an arrangement of precision optics F-theta-lens. The atmosphere inside the build chamber was stringently maintained inert by the circulation of argon. The samples were fabricated using a layer thickness of 30 μm and a hatch spacing of 90 μm using a variety of scanning strategy, as shown in Figure 4.

The samples were fabricated using a constant scan speed of 425 mm/s and a laser power of 85 W. These parameters were chosen following an extensive review work [44]. Thin-layered samples of approximately 1.0 to 1.5 mm thickness in the build direction were fabricated against the proposed thickness of 25 mm. The printed samples were separated from the build plate by applying shear force at the base of prints using a scrapper and gentle tapping. However, issues with delamination and excessive warping (caused due to residual internal stresses owing to uneven cooling of printed samples) prevented the fabrication of thicker samples.

### 2.4. Metallographic Preparation

A Struers automatic grinder and polisher were used to grind and polish the primitive structures. The samples were hot-mounted before grinding at a pressure of 30 N and temperature of 180 °C in a Struers hot press using phenolic resin for 7 min. Silicon carbide grinding paper of successively decreasing grit sizes was used to fine grind the mounted samples. Using the slurry of suspended diamond abrasives of varying sizes (3–0.04 μm), the ground samples were finely polished. Colloidal silica suspension (0.02 μm) slurry was used at the final stage in order to obtain mirror-like surface polish. Sonication and plasma cleaning methods were used to remove the debris and contaminants on the surface of the polished samples. In order to obtain clear micrographs, contrast in the different microstructural features present in the samples was created using an etchant consisting of a diluted solution of hydrofluoric acid and nitric acid.

### 2.5. Microstructural Characterization

The microstructure of the powder and the printed samples was observed using a Schottky field emission SEM (JEOL JSM 7610F) at room temperature. Accelerating voltages of 3–10 keV were used for imaging. X-ray spectroscopy (EDX) of the powder and other samples was performed using an Oxford Instrument device at an accelerating voltage of 30 keV. The phase composition of the samples was determined using a Bruker Phase D2 diffractometer with Cu Kα radiation filter and an operating voltage of 30 kV. A step size of 0.5° was used for scanning with a 2θ variation from 5° to 90° at room temperature. The PDF-4 database was used to analyze the X-ray diffraction patterns. 

## 3. Results and Discussion

AM of the NiTi primitive lattices was carried out using different sets of laser process parameters, as listed in Table 1. 

Variation in relative density and scan strategy was investigated, maintaining fixed values for the process parameters.

### 3.1. Macroscopic Analysis

**Figure 5:**
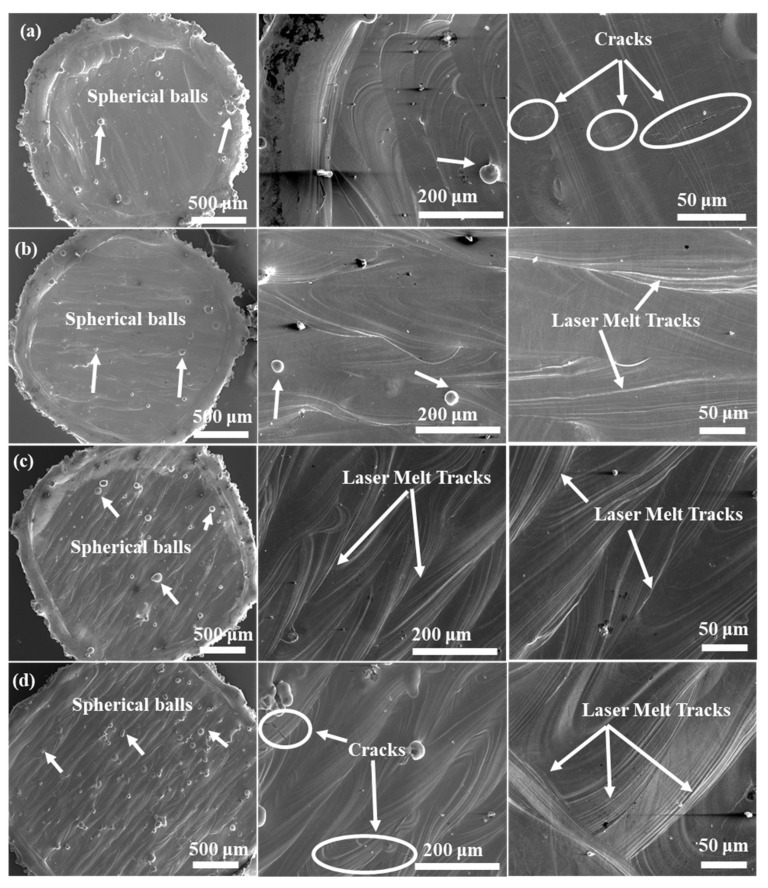
SEM images showing macroscopic features of LPBF fabricated primitive lattice structures with varying relative densities (**a**) RD30, (**b**) RD40, (**c**) RD50, and (**d**) RD60.

Figure 5 shows the scanning electron micrographs (SEM) of LPBF fabricated primitive TPMS structures with varying relative densities. 

**Figure 6:**
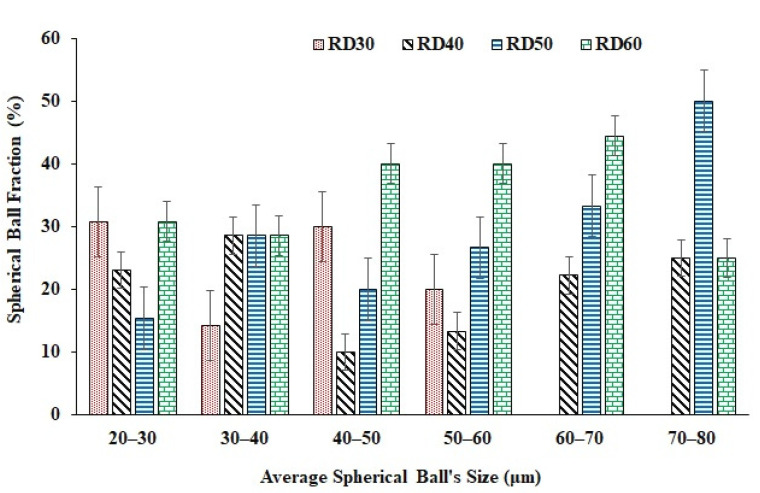
LBPF-manufactured samples showed the formation of spherical balls. The bar chart shows the distribution of average particle sizes of the spherical balls formed as a function of relative density.

Preliminary macroscopic analysis shows the formation of spherical balls on the surface of the samples, as well as the presence of cracks. The laser tracks can also be clearly observed on the top surface of the samples, which have been marked in the figure for illustration. Several theories have been proposed in the literature [50,51,52] to explain the balling phenomenon commonly observed in samples fabricated using LPBF. In particular, the small size of the molten pool during laser melting is reported to cause solidification into spherical imperfections rather than forming a continuous surface. The presence of oxygen also promotes balling as it affects the wetting of the molten pool to the previous layer, thereby favoring ball formation rather than continuous lines over sublayers. Different shapes and sizes of balls have been reported, such as ellipsoidal balls and spherical balls [50]. The spattering of melt pool may also contribute to balling on the surfaces of the printed surfaces. In the present study, primarily spherical balls of varying sizes have been found, as shown in Figure 6. 

With the increase in relative density, balling was found to increase. Sample RD60 shows the formation of balls of higher average sizes compared to the other samples, implying that higher relative densities may favor the formation of larger balls (greater than 50–60 μm). In contrast, the sample of 30% relative density showed the formation of small balls of average sizes less than 50–60 μm. In addition to balling, small cracks of various lengths and densities were observed in the samples. These are likely due to the rapid melting and solidification of the metal powder during LPBF, in which the cooling rate of the molten pool may reach up to 10^8^ K/s. Such a high rate results in large temperature gradients in the solidified part, which, in turn, leads to potentially high residual stress [53,54]. The combination of high residual stress and large temperature gradient leads to the initiation and propagation of cracks in the fabricated parts. It was observed that samples with relative densities between 40% and 50% were less prone to cracking compared to those with 30% or 60% density. The correlation between density and propensity for cracking, therefore, appears to be a non-monotonic trend. 

In terms of scan strategy, variations can be affected by controlling a range of process variables, including scan time, hatch spacing, scanning directions, scanning vector length, scanning vector rotation angle, and scanning sequence. In the present study, the scanning vector rotation angles of 0°, 45°, and 90° were selected to investigate their influence on the microstructure of the fabricated NiTi TPMS structures. 

**Figure 7:**
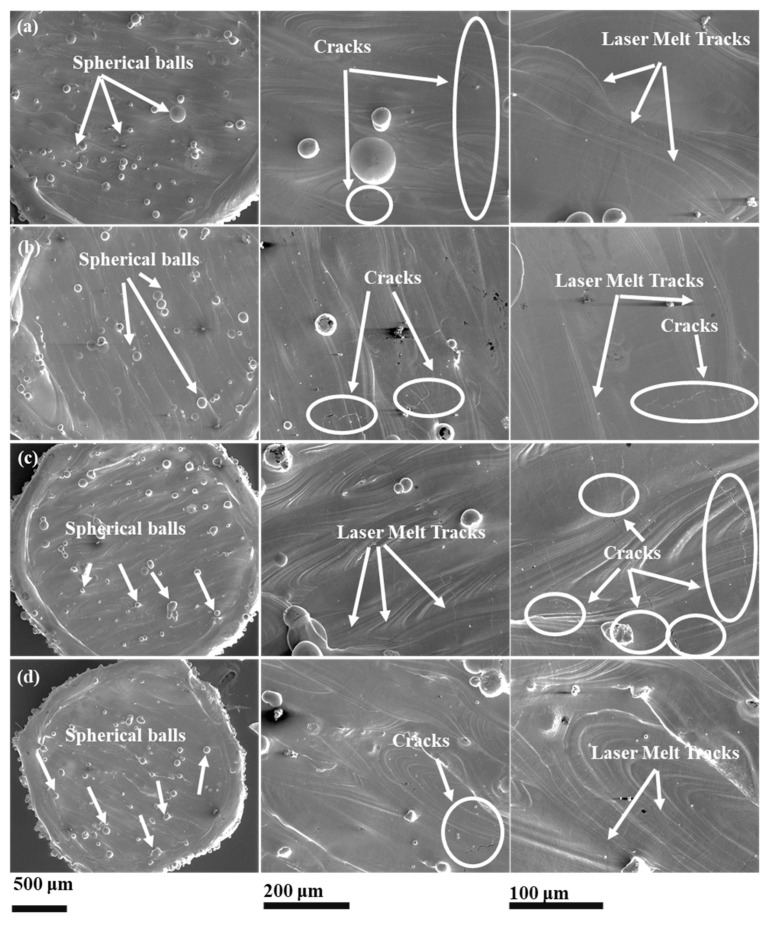
SEM images of primitive lattice structures additively manufactured using different scan strategies (**a**) S1, (**b**) S2, (**c**) S3, and (**d**) S4.

**Figure 8:**
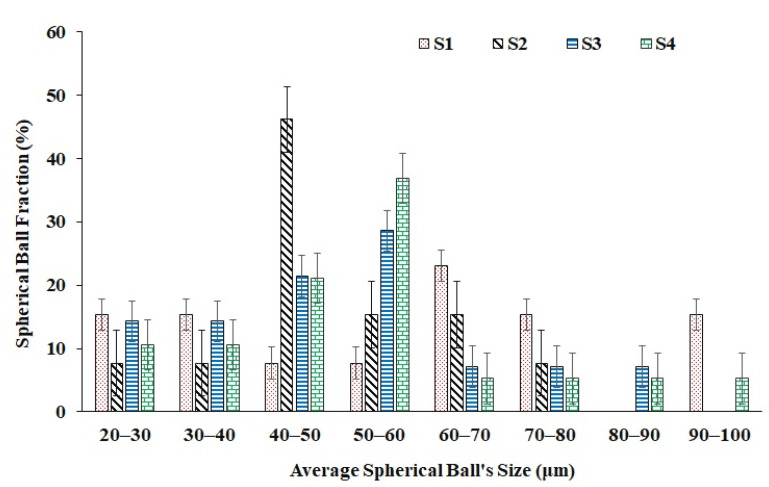
LBPF manufactured samples showed formation of spherical balls. The bar chart shows the distribution of average particle sizes of the spherical balls formed as a function of scan strategy.

Figure 7 shows the electron micrographs of the primitive lattice samples fabricated using different scanning strategies. The macroscopic observation reveals the presence of balls and cracks on the external surfaces of the produced parts. As discussed earlier, balling imperfections are typically associated with the LPBF methods and can be found often. Laser process parameters optimization can be one of the methods to reduce or eliminate the balling. All samples, printed with different scan strategies, showed crack formation, as shown in Figure 8. However, scan strategies could influence the crack formation and their characteristics in LPBF-manufactured samples, as reported elsewhere [43,55]. As evident from Figure 7 and Figure 8, the inclined scan strategy has increased the balling in the fabricated parts as compared to other scan strategies. However, the average particle sizes remained between 40 and 60 μm.

### 3.2. Microscopic Analysis

**Figure 9:**
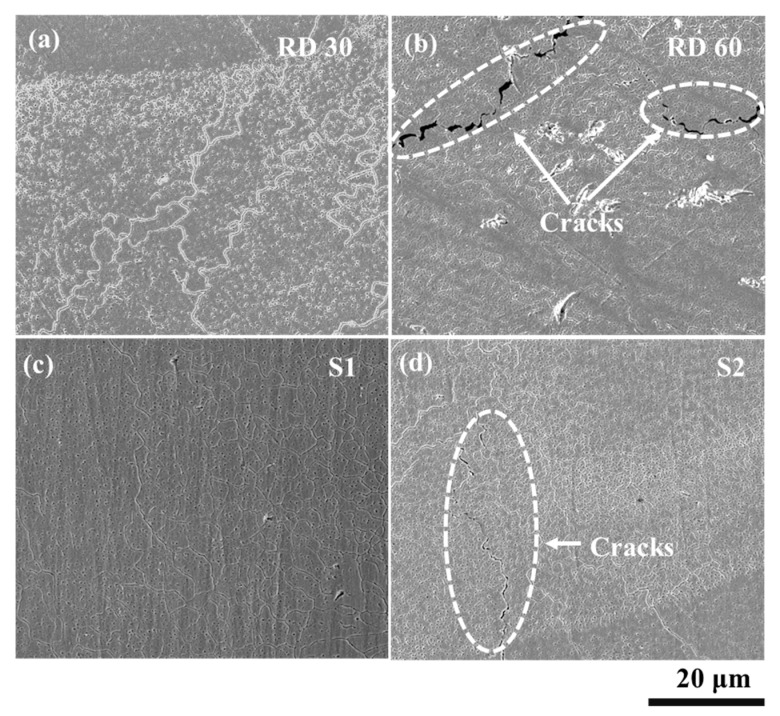
SEM images showing the microstructure of LPBF manufactured primitive lattices NiTi structures with varying relative densities and scan strategies (**a**) RD30, (**b**) RD60, (**c**) S1, and (**d**) S2.

Figure 9 shows the SEM images of LPBF manufactured NiTi TPMS lattice samples. 

Figure 9a,b shows the fabricated samples with relative densities of 30% and 60%, respectively. Figure 9c,d shows the samples fabricated using different scan strategies, namely parallel strategies with 90° and 0° rotation, respectively. The microstructures show a wide range of grain shapes, sizes, and orientations. 

In Figure 9b,d, cracks are seen to form along grain boundaries with crack lengths and opening significantly higher in Figure 9b. The formation of cracks, in Figure 9d in particular, is accompanied by strong grain size gradients. Such variation is indicative of non-uniform solidification during the fabrication processes. Changes in grain morphology, in addition to size, are also clearly visible in Figure 9c, with elongated grains near the bottom right transitioning toward more regular configurations into the bulk. Further transition from regular equiaxed grains to larger grains can be observed near the top left of the same micrograph. The inhomogeneity in the grain structures indicates a non-uniform cooling rate during solidification of the molten pool and has a strong influence on the mechanical properties of the fabricated samples. Improved homogeneity may be achievable through the optimization of the LPBF process parameters. The imperfections such as balling, cracks, and heterogenous microstructure can be deleterious to mechanical behaviors of the AM products and, therefore, need to be thoroughly investigated in order to inhibit their formations. 

## 4. Conclusions

NiTi Schwarz primitive TPMS sample layers were manufactured using LPBF. The samples were fabricated considering varying relative densities and scanning strategies in order to study the microstructure of the built parts. Other laser process parameters, namely scan speed, hatch spacing, layer thickness, and laser power, were not varied during fabrication. The obtained samples were metallographically prepared, and their microstructural features were examined using SEM. Balling was observed in all the samples. However, it was most pronounced at the highest density of 60% without following a monotonic trend over the range of densities considered in this work. Moreover, balling was most noticeable in samples printed using an inclined scan strategy, and it was accompanied by a considerable formation of mostly intergranular cracks. No uniformly increasing or decreasing pattern was noted in the balling phenomenon vis-à-vis scan strategies. However, the parallel scanning strategy with interlayer rotation of 90° is considered more beneficial for obtaining even temperature distribution during AM, resulting in less residual stresses. Microstructure, on the other hand, indicated a non-uniform solidification rate, however, strict trends with relative density and scanning strategy could not be demonstrated. Balling on the surface of printed structures can also be due to the spattering of melt pool. The present work, therefore, suggests that the most commonly reported approach for optimizing LPBF parameters for fabricating nitinol, which relies on considering those parameters immediately influencing the laser energy density supplied to the powder, may need to be enriched with considerations of scan angle and overall structural topology and density when seeking LPBF fabricated parts of optimal quality. 

## Figures and Tables

**Table 1:** Laser process parameters used during LPBF of primitive lattice structures.

	Relative Density (%)	Scan Strategy	Laser Power (W)	Scan Speed (mm/s)	Hatch Spacing (μm)	Layer Thickness (μm)
**RD30**	30	Parallel with 90° rotation	85	425	90	30
**RD40**	40	Parallel with 90° rotation	85	425	90	30
**RD50**	50	Parallel with 90° rotation	85	425	90	30
**RD60**	60	Parallel with 90° rotation	85	425	90	30
**S1**	30	Parallel with 90° rotation	85	425	90	30
**S2**	30	Parallel with 0° rotation	85	425	90	30
**S3**	30	Inclined at 45°	85	425	90	30
**S4**	30	Spiral	85	425	90	30

## Data Availability

Not applicable.
